# Inhibition of phosphorylated c-Met in rhabdomyosarcoma cell lines by a small molecule inhibitor SU11274

**DOI:** 10.1186/1479-5876-9-64

**Published:** 2011-05-16

**Authors:** Jinxuan Hou, Jixin Dong, Lijun Sun, Liying Geng, Jing Wang, Jialin Zheng, Yan Li, Julia Bridge, Steven H Hinrichs, Shi-Jian Ding

**Affiliations:** 1 Department of Pathology and Microbiology, University of Nebraska Medical Center, Omaha, 68105 USA; 2 Department of Oncology, Zhongnan Hospital of Wuhan University, Wuhan, 430071 China; 3 Eppley Cancer Institute, University of Nebraska Medical Center, Omaha, 68105 USA; 4 Department of Pharmacology and Experimental Neuroscience, University of Nebraska Medical Center, Omaha, 68105 USA

## Abstract

**Background:**

c-Met is a receptor tyrosine kinase (RTK) that is over-expressed in a variety of cancers and involved in cell growth, invasion, metastasis and angiogenesis. In this study, we investigated the role of c-Met in rhabdomyosarcoma (RMS) using its small molecule inhibitor SU11274, which has been hypothesized to be a potential therapeutic target for RMS.

**Methods:**

The expression level of phosphorylated c-Met in RMS cell lines (RD, CW9019 and RH30) and tumor tissues was assessed by phospho-RTK array and immunohistochemistry, respectively. The inhibition effects of SU11274 on RMS cells were studied with regard to intracellular signaling, cell proliferation, cell cycle and cell migration.

**Results:**

A high level of phosphorylated c-Met was detected in 2 alveolar RMS cell lines (CW9019 and RH30) and 14 out of 24 RMS tissue samples, whereas relatively low levels of phospho-c-Met were observed in the embryonic RMS cell line (RD). The small molecule SU11274 could significantly reduce the phosphorylation of c-Met, resulting in inhibition of cell proliferation, G1 phase arrest of cell cycle and blocking of cell migration in CW9019 and RH30 cell lines.

**Conclusion:**

These results might support the role of c-Met in the development and progression of RMS. Furthermore, the inhibitor of c-Met, SU11274, could be an effective targeting therapy reagent for RMS, especially alveolar RMS.

## Background

Rhabdomyosarcoma (RMS) is the most common soft tissue tumor in childhood, accounting for up to 50% of all soft tissue sarcomas [1]. While in adults, RMS represents about 15-20% of all soft tissue sarcomas [2]. There are two main histologically distinct subtypes of RMS: embryonal RMS (ERMS) and alveolar RMS (ARMS) [3]. ERMS is composed of spindle-shaped cells with a stromal rich appearance and occurs mainly in the head and neck region. It is the most frequently diagnosed variant with a generally good prognosis and presents early with an onset around the age of 2-5 years [3,4]. In contrast, ARMS consists of small, round, densely packed cells and occurs more often in the trunk and extremities. ARMS is primarily diagnosed in adolescents and is associated with a poor prognosis as patients often present with metastatic disease [5]. Chemotherapy is the most common therapeutic option for RMS. The regimens are typically based on variations of the well-established vincristine, actinomycin D and cyclophosphamide, or a combination of the alkylating agent ifosfamide with carboplatin and the topoisomerase II etoposide [6]. Patients with metastatic stage IV ERMS and those with ARMS continue to face a poor prognosis because of diminished tumor response to current chemotherapeutic options [5,7]. Therefore, the development of novel therapeutic strategies for these RMS patients is urgently needed.

Receptor tyrosine kinases (RTKs) are key regulators of critical cellular processes such as cell growth, differentiation, neovascularization and tissue repair. In addition to their importance in normal physiology, aberrant expression of certain RTKs has been implicated in the development and progression of many types of cancer. These RTKs have emerged as promising drug targets for cancer therapy [8]. RTKs can initiate tumor growth (Bcr-abl in chronic myelogenous leukemia [9,10]) or sustain tumor survival (EGFRmut in non-small cell lung carcinoma [11,12] and c-Kit in gastrointestinal stromal tumors [13]). Inhibiton of RTKs by small, targeted molecules has exhibited significant clinical benefit in cancer patients in several selected circumstances.

The present work aims to identify such therapeutic targets for RMS. Based on the data from phospho-receptor tyrosine kinase (p-RTK) array, a high expression level of phosphorylated c-Met was observed in 3 RMS cell lines. c-Met is the receptor of hepatocyte growth factor/scatter factor (HGF/SF). There is now considerable evidence suggesting that aberrant c-Met/HGF/SF signaling plays a major role in tumorigenesis, invasion, and metastatic spread of many human tumors, resulting from mutation or over-expression of the c-Met proto-oncogene and/or its ligand [14-16].

We hypothesized that c-Met signaling played a key role in RMS oncogenic signaling and that optimized therapy targeting c-Met would be effective as a treatment strategy. Recently, a small molecular c-Met inhibitor, SU11274, has been developed and shown to inhibit c-Met phosphorylation and c-Met-dependent motility, invasion, and proliferation in lung cancers *in vitro *[17,18]. Furthermore, it could abrogate HGF-induced phosphorylation of c-Met and its downstream signaling including phospho-AKT, phospho-ERK1/2, phospho-S6 kinase, and phospho-mTOR (mammalian target of rapamycin) [17]. In the current study, we employed and evaluated the effect of SU11274 on proliferation, cell cycle and migration of RMS cells.

## Methods

### Reagents and antibodies

SU11274 was obtained from EMD Biosciences (San Diego, USA). Hepatocyte growth factor (HGF) was purchased from R&D Systems (Minneapolis, USA). Antibodies against phospho-c-Met (pY1234/1235), total c-Met, phospho-STAT3 (Tyr705), total STAT3, phospho-AKT (S473), total AKT, phospho-ERK1/2 (T202/204) and total ERK1/2 were obtained from Cell Signaling Technology (Danvers, USA). Myogenin was purchased from Santa Cruz Biotechnology (Santa Cruz, CA).

### Cell lines and cell culture

RMS cell lines (RD and RH30) and the normal muscle cell line (HASMC) were purchased from American Type Culture Collection (ATCC). The CW9019 cell line was kindly provided by Frederic G. Barr (School of Medicine, University of Pennsylvania). Cells were grown in Dulbecco's Modified Eagle Medium (DMEM) (RD, CW9019 and HASMC) and RPMI1640 medium (RH30) (Mediatech, Manassas, USA) supplemented with 10% fetal bovine serum (FBS) and 1% penicillin/streptomycin (Gibco, Carlsbad, USA). The cells were cultured in a humidified atmosphere at 37°C in 5% CO_2_.

### Patients and tissue samples

A tumor tissue microarray was obtained from US Biomax, Inc (Rockville, MD, USA) and consisted of 18 RMS tumor tissues and 3 normal muscle tissues. These patients included 8 males and 8 females with a median age of 40 years (range: 18-91). 6 additional ARMS tissues were obtained from Zhongnan Hospital of Wuhan University (Wuhan, China). There were 3 males and 3 females with a median age of 37 years (range: 13-61). Written informed consent was obtained from the patients and the study protocol was approved the Institutional Review Board (IRB) at the University of Nebraska Medical Center (UNMC, Omaha, USA).

### Phospho-RTK array

A human p-RTK array kit (R&D Systems, Minneapolis, USA), which has a greater sensitivity than immunoprecipitation analysis, was used to simultaneously detect the relative tyrosine phosphorylation levels of 42 different RTKs in RMS cell lysates. Each array contained duplicate validated control and capture antibodies for specific RTKs. RMS cells were cultured for 24 h in serum-free medium at 37°C in a humidified atmosphere of 5% CO_2 _in air, and then immediately placed on ice, washed twice with chilled PBS, and isolated using chilled lysis buffer (20 mM Tris-HCl, pH 8.0, 150 mM NaCl, 1% NP-40, 2.5 mM EDTA, 1 mM sodium orthovanadate, 10% glycerol, 10 μg/ml aprotinin, 10 μg/ml leupeptin). Total protein concentration was quantitated using a Coomassie Brilliant Blue (CBB) assay kit (Pierce, Rockford, USA). RTK array analysis was performed according to the manufacturer's protocol. In brief, the array membrane was blocked and incubated with cell lysates for 2 h, then treated with HRP conjugated anti-phospho-tyrosine antibody for 2 h at room temperature. The membrane was developed with ECL detection reagent (Pierce, Rockford, USA), and RTK spots were visualized using Kodak XAR film (Fisher Scientific, Houston, USA).

### Immunohistochemistry

The tissue slides were treated with xylene to remove paraffin, then with a decreasing gradient of ethanol. Then the slides were pre-treated in 0.01 M citrate buffer (pH 6.0) and heated in a microwave oven (98°C) for 10 min. Endogenous peroxidase was blocked for 20 minutes with a 3% hydrogen peroxide solution. The slides were processed for detection of phospho-c-Met expression using the primary antibody for phospho-c-Met (Tyr1234/1235, 1:160 dilution) and a secondary antibody (HRP-conjugated goat anti-rabbit IgG) in 10% goat serum. The reaction products were visualized with diaminobenzidine (DAKO, Denmark). For the tissue array, normal muscle tissues were included as negative controls and duplicate specimens were included in the array. For tissue slides, the primary antibody was replaced with IgG for a negative control. All slides were independently analyzed by two investigators. The staining score was calculated from the staining intensity and percentage of positive staining cells. The staining intensity was scored as 1 (very weak), 2 (weak), 3 (moderate) and 4 (intense). The positive rate score was 0 (0-10%), 1 (10-30%), 2 (30-50%), 3 (50-75%) and 4 (> 75%). The score of each slide was the sum of intensity and positive rate scores. The staining results were categorized as low expression (the score ≤ 3) and high expression (the score > 3).

### Proliferation/cell survival assay

RMS cells were plated at 1 × 10^4 ^cells/well in 96-well plates and allowed to adhere overnight. Serum-starvation was performed for 6 h. Then the cells were treated with SU11274 at the indicated concentrations for 72 h with or without the presence of HGF (10 ng/ml). Dimethyl sulfoxide (DMSO) was added to the control with the same volume. The viability of the cells was determined by the MTT proliferation/viability assay (Invitrogen, Carlsbad, USA) according to the manufacturer's instruction.

### Western blot

Western blot analyses were performed to detect specific phosphorylation of c-Met and other signaling molecules via HGF and inhibition of phosphorylation with SU11274. RMS cells were deprived of growth factors by incubating them in serum-free medium for 6 h followed by treatment with SU11274 (5 μM) or DMSO for 24 h. Then the cells were either left untreated or they were treated with HGF (10 ng/ml) for 7.5 min. For the lysate preparation, cells were first washed with PBS and lysed in 2× sodium dodecyl sulphate sample buffer (100 mM Tris-HCl pH6.8, 200 mM DTT, 4% SDS, 20% glycerol and 0.2% bromophenol blue). Then the cell lysates were separated on 8% or 10% SDS-PAGE. Proteins were transferred to an immobilization membrane (Millipore, Billerica, USA) and immunoblotted using enhanced chemiluminescence (ECL; GE Healthcare Life Sciences, Piscataway, USA).

### Cell cycle analysis

RMS cells were treated with SU11274 (5 μM) or an equal volume of DMSO for 24 h. Cells were collected and stained with propidium iodide according to the standard protocol of the FACS core facility (UNMC, Omaha, USA). The cell cycle was analyzed by a BD FACSCalibur flow cytometer (BD Biosciences, San Jose, USA) with CellQuest Pro software (BD Biosciences, USA).

### Wound healing assay

RMS cells were seeded at a density of 5 × 10^5 ^cells per well in a 6-well plate and grown overnight to confluence in complete medium. The cells were serum starved for 6 h and then treated with DMSO or SU11274 (5 μM) for 24 h, respectively. The monolayer was scratched with a pipette tip and washed with phosphate buffered saline (PBS) to remove floating cells. The scrape was monitored and photographed after 24 h incubation.

### Trans-well assay

Trans-well motility assays were performed utilizing 8 μm pore, 6.5 mm polycarbonate trans-well filters (Costar, Cambridge, USA) according to the standard protocol. In brief, RMS cells were plated onto the upper well of the trans-well previously coated with 50 μl of Matrigel Basement Membrane Matrix (BD, Franklin Lakes, USA) and then treated with DMEM or SU11274 (5 μM) for 24 h. The noninvasive cells on the upper surface of the membrane were removed with a cotton swab. Cells that attached to the lower surface of the membrane and migrated through the Matrigel matrix were fixed with glutaraldehyde, stained with cresyl violet, solubilized in 10% acetic acid solution, and quantified by spectrophotometric analysis (570 nm).

## Results

### Expression of phosphorylated RTKs in RMS

**Figure 1:**
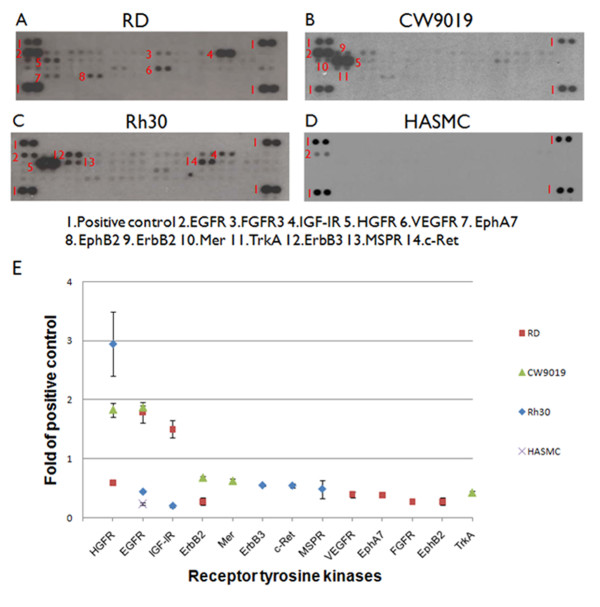
**Expression of phosphorylated RTKs in RMS cell lines**. Multiple RTKs are detected in RMS cell lines RD (A), RW9019 (B), RH30 (C) and normal muscle cell line HASMC (D). Whole-cell extracts were incubated on RTK antibody arrays and phosphorylation status was determined by subsequent incubation with anti-phosphotyrosine horseradish peroxidase. Each RTK is spotted in duplicate and the pairs of dots in each corner are positive controls. Each pair of positive RTK dots is denoted by a red numeral, with the identity of the corresponding RTKs listed below the arrays. E, thirteen overexpressed RTKs were semi-quantified with Image J software (NIH, USA).

To evaluate the expression of phosphorylated RTKs in RMS, the phospho-RTK array was used with three RMS cell lines RD (ERMS), CW9019 (ARMS), RH30 (ARMS) and one normal muscle cell line HASMC (Figure 1A-D). Thirteen RTKs were detected in the RMS cell lines, including HGFR (c-Met), epidermal growth factor receptor (EGFR), insulin growth factor-I receptor (IGF-IR), ErbB2, ErbB3, c-Ret, MSPR, VEGFR, Mer, EphA7, FGFR, EphB2 and TrkA. The expression levels of RTKs for each cell line are shown in Figure 1E. The phosphorylation level of c-Met was significantly higher in ARMS cell lines (CW9019 and RH30) and slightly higher in the ERMS cell line (RD) in comparison with the normal muscle cell line HASMC.

### Expression of phosphorylated c-Met in tissue samples

**Figure 2:**
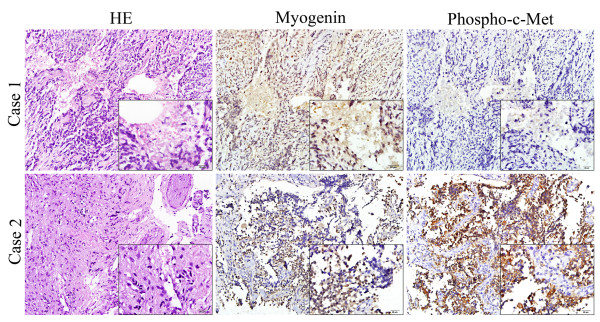
**Analysis of the expression and localization of phosphorylated c-MET in RMS tissue samples**. Represent images of HE staining and IHC staining of myogenin and phospho-c-Met were shown. Case 1 is phospho-c-Met negative whereas case 2 is phospho-c-Met positive. Positive staining of phospho-c-Met was observed in both membrane and cytoplasm. Magnification, ×100 and ×400 (inserts).

To evaluate the possible clinical significance in RMS, the expression of phosphorylated c-Met was assessed in 24 RMS tissues and 3 normal muscle tissues using immunohistochemistry. Phosphorylated c-Met protein was localized in both the membrane and the cytoplasm (Figure 2). The results showed that phospho-c-Met was over-expressed in 14 of 24 (58.3%) RMS tissues, including 5 ERMS, 7 ARMS and 2 Pleiomorphic RMS (Table 1). None of the normal muscle tissues stained positively.

**Table 1:** Summary of phosphorylated c-Met expression in RMS tissue samples (n = 24)

Histology type	Low expression (n/%)	High expression (n/%)
ERMS	8/33.3%	5/20.9%
ARMS	1/4.2%	7/29.2%
Pleomorphic RMS	1/4.2%	2/8.3%

### Effect of SU11274 on the proliferation and c-Met signaling pathway

**Figure 3:**
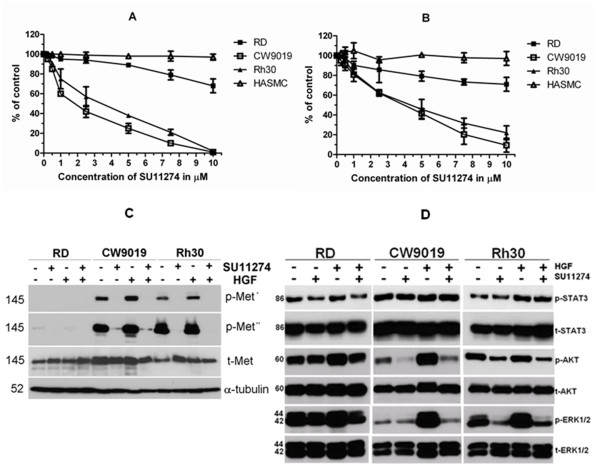
**Inhibition effect of SU11274 on proliferation and intracellular signaling in RMS cells**. A and B, Cells were plated in 96-well plates and allowed to adhere overnight followed by treatment with the indicated concentrations of SU11274 without (A) or with (B) 10 ng/ml HGF. MTT proliferation/viability assay was performed after 72 h treatment. Data represent mean ± SD for triplicate independent experiments. C and D, expression of c-Met (C) and its downstream kinases (D) modulated in three RMS cell lines after treatment with 5 μM SU11274 for 24 h. RMS cells were pre-starved and stimulated with 10 ng/ml of HGF for 7.5 min. Cells were harvested and immunoblotted using phospho-specific antibodies against phospho-c-Met (pY1234/1235), phospho-STAT3 (Tyr705), phospho-AKT (S473) and phospho-ERK1/2 (T202/204). *short exposure; **long exposure.

To determine whether c-Met was a potential therapeutic target, the high specific c-Met inhibitor SU11274 was used to block c-Met function in 3 RMS cell lines and 1 normal muscle cell line, HASMC. In CW9019 and RH30 cell lines, which expressed high levels of phospho-c-Met, the IC_50 _of SU11274 was 2.5 μM; whereas in the RD cell line, which expressed a lower level of phospho-c-Met, the IC_50 _was over 7.5 μM. The effect of SU11274 on the normal muscle cell line, HASMC, was mild as shown in Figure 3A. The results indicated that the cytotoxicity of SU11274 might be correlated with the expression level of phosphorylated c-Met. When the cells were treated with SU11274 in the presence of HGF (10 ng/ml) (Figure 3B), more cells survived than when HGF was omitted (Figure 3A). The results suggested that HGF could protect cells from the cytotoxicity of SU11274, which might be due to an increased phosphorylation level of c-Met caused by HGF.

We tested the effects of SU11274 and HGF on the phosphorylation level of c-Met in RMS cell lines. The results showed that treatment with HGF increased the autophosphorylation of c-Met at the activation loop site phospho-epitope (pY1234/1235). Whereas, SU11274 significantly reduced phosphorylation of the above tyrosine residues at the activation site (Figure 3C).

Met kinase autophosphorylation was reduced on sites that have been shown to be important for the activation of pathways involved in cell proliferation, differentiation, survival, motility and death, especially the phosphoinositide-3-kinase (PI3K) pathway and the mitogen activated protein kinase (MAPK) pathway. We then analyzed the phosphorylation level of c-Met, and its downstream signaling molecules AKT, STAT3 and ERK1/2 with or without SU11274 treatment (Figure 3D). We observed that the phosphorylation of c-Met, AKT and ERK1/2 was abolished by SU11274 in both HGF-induced and non-induced conditions in CW9019 and RH30 cell lines, whereas the effect of SU11274 was weak in the RD cell line. This could be correlated with the expression level of phosphorylated c-Met. However, the phosphorylation level of STAT3 was not influenced by SU11274 in any of the three cell lines. The results indicated that phosphorylation of c-Met could activate the PI3K and MAPK signaling pathways but not the STAT pathway.

### Effect of SU11274 on cell cycle and apoptosis in RMS cell lines

The effect of SU11274 on the cell cycle and apoptosis was evaluated by flow cytometry. Cells were treated with DMSO or SU11274 (5 μM) and the different phases of cell cycle distribution were determined. The percentage of cells in G1 phase increased significantly whereas the percentage of cells in S phase and G2/M phase decreased (Table 2). In addition, there was also an increase in apoptosis after SU11274 treatment. These data indicated that SU11274 could induce G1 cell cycle arrest and apoptosis, and both events in combination might contribute to the reduced cell growth of SU11274 treated RMS cells.

**Table 2:** The percentage of cells in different cell cycle phases

% of cells in phase	RD			CW9019			RH30		
			
	Control	SU11274	SD	Control	SU11274	SD	Control	SU11274	SD
G_1_	51.35	59.40	0.5	50.48	66.16	0.7	53.57	68.40	0.8
S	22.65	25.40	0.2	23.16	21.15	0.3	27.87	19.04	0.2
G_2_/M	25.99	15.20	0.1	26.36	12.68	0.3	18.56	12.56	0.1
Apoptosis	0.16	2.84	0.02	0.10	5.12	0.07	0.16	4.61	0.03

### SU11274 inhibited cell motility in RMS cell lines

**Figure 4:**
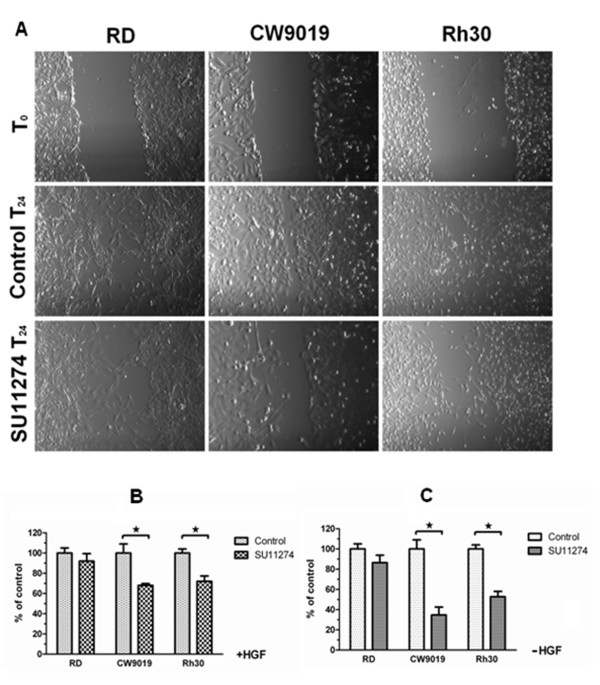
**SU11274 blocked motility in RMS cell lines**. A, SU11274 inhibited wound healing in RMS cell lines. RMS cells were seeded at a density of 5 × 10^5 ^cells per well in a 6-well plate and grown overnight to confluence in serum containing media. The cells were serum starved for 6 h and pretreated with DMSO or SU11274 (5 μM), respectively. The monolayer was scratched with a pipette tip and washed with 1 × PBS to remove floating cells. The scrape was monitored and photographed after 24 h. B and C, SU11274 inhibited trans-well migration with (B) or without (C) presence of HGF. Data represent mean ± SD for triplicate independent experiments. * *P *< 0.05.

Cell motility was evaluated using the *in vitro *wound healing/scratch assay (Figure 4A) and the trans-well assay (Figure 4B and 4C). The results from the scratch assay showed that the motility of CW9019 and RH30 cell lines was inhibited by SU11274, while the RD cell line motility was not inhibited. The RD cell line grew nearly to confluence like the SU11274 untreated controls. This might be because the effectiveness of SU11274 depends on the phosphorylation level of c-Met. However, inhibition of cellular proliferation may also contribute to the effects seen in the scratch assay. We also performed trans-well assays to quantify the effect of SU11274 on cell motility. The results showed that SU11274 significantly inhibited migration of CW9019 and RH30 cells, while there was little inhibition in RD migration. In addition, studies were performed in the presence of HGF which served as the ligand for c-Met. The results showed that HGF treatment reduced SU11274 inhibition in CW9019 and RH30 cells but had little effect in RD cells, which was consistent with the observations in the cell survival assay.

## Discussion

The successful development of molecular agents that will inhibit tumor growth is dependent on the identification of targets that are directly involved in tumorigenesis and development. Receptor tyrosine kinases (RTKs) are key regulators of critical cellular processes which are activated through phosphorylation or over-expression in the development and progression of many types of cancer. They have emerged as promising drug targets for cancer therapy.

In the current study, we identified thirteen phosphorylated RTKs that were over-expressed in three RMS cell lines RD (ERMS), RW9019 (ARMS) and RH30 (ARMS) using the phospho-RTK array (Figure 1). Importantly, other groups have also reported the over-expression of several RTKs in these cell lines. For instance, it has been reported that IGF-IR is highly expressed in RD and RH30 cell lines and could be a potential therapeutic target for RMS both *in vitro *and *in vivo *[19]. In addition, EGFR is highly expressed in ERMS tumor tissue [20-22]. Expression of ErbB2 is more prevalent in ARMS tumor tissue where it is found in the majority of RMS tumors of the head and neck [23]. ErbB3 is over-expressed in RMS cells and may play a role in regulating differentiation [24].

We have focused on another RTK, HGFR/c-Met, mainly due to its over-expression in all three RMS cell lines, which was consistent with previous reports [25,26]. We found that 14 of 24 (58.3%) RMS tumor tissues showed high expression level of phosphorylated c-Met (Figure 2). Over-expression of HGF and c-Met has been reported to correlate with increased aggressiveness of tumors and a poor prognosis in cancer patients [27]. In tumor cells, c-Met activation triggers a diverse series of signaling cascades resulting in cell growth, proliferation, invasion, and protection from apoptosis [28,29]. Data from cellular and animal tumor models suggest that the underlying biological mechanisms for tumorgenicity of c-Met are achieved in three different ways: (i) with the establishment of HGF/c-Met autocrine loops; (ii) via c-Met or HGF over-expression; and (iii) in the presence of kinase-activating mutations in the c-Met receptor coding sequence [28,30-32]. There were no activating mutations in the tyrosine kinase region of the c-Met receptor in the RMS cell lines used in our experiments (RD, CW9019 and RH30), and no functional autocrine regulatory loops were present. One explanation for higher expression of c-Met in these RMS cells is modulation by the PAX3/7-FOXO1 fusion gene [33]. In this study, both ARMS cells lines have the PAX3-FOXO1 (RH30) and PAX7-FOXO1 (CW9019) translocations and express more phosphorylated c-Met than the PAX-FOXO1-negative ERMS cell lines (RD).

It has been proposed that targeting c-Met by novel biological agents will inhibit tumor progression at the molecular level. Recently, cell proliferation *in vitro *and tumor burden in mouse xenograft models were decreased by targeted knockdown of c-Met using siRNA in human RMS cell lines [34,35]. In order to improve clinical application of this concept, several different strategies are being explored, including the development of competitors of c-Met/HGF, monoclonal antibodies directed against HGF and c-Met, and small-molecule tyrosine kinase inhibitors directed against c-Met [8].

We hypothesized that inhibition of phosphorylation on c-Met with the specific, small-molecular inhibitor SU11274 might induce anti-tumor effects. We examined the cytotoxicity of SU11274 without and with HGF treatment on three RMS cell lines and one normal muscle cell line (Figure 3A and 3B). SU11274 inhibited the proliferation of RMS cells that exhibited high levels of phosphorylated c-Met in a dose dependent manner. This suggested that the inhibitory effects mgithe be associated with c-Met driven proliferation of RMS cell lines. In addition, the phosphorylation levels of AKT and ERK 1/2 downstream in the c-Met signaling pathways were almost completely abolished when phosphorylated c-Met was blocked by SU11274 in RMS cell lines (Figure 3C and 3D), agreeing with the results from previous studies in several types of malignancies [17,36,37]. The results indicated that the advantage of c-Met inhibition was that multiple pathways were silenced by a single upstream intervention.

RMS cells, especially ARMS, show strong directional chemotaxis [33]. Accordingly, we performed wound healing and trans-well assays to evaluate the effect of SU11274 on RMS cell migration. Both of the results showed that the migration of CW9019 and RH30 cells was significantly inhibited by SU11274 compared with RD cells, which indicated that the ability of SU11274 to block cellular migration might correlate with the expression level of phospho-c-Met (Figure 4). There are several RD derived clones which express quite high levels of phospho-c-Met. Therefore, the effects of SU11274 treatment on these RD clones may be as significant as we observed in CW9019 and RH30 cells. In addition, we found that SU11274 treatment induced G1 phase arrest and apoptosis in RMS cells (Table 2), which was also observed in other tumors such as NSCLC [17], melanoma [36] and head and neck squamous cell carcinoma [37].

## Conclusions

We have shown that phosphorylated c-Met was over-expressed and activated as a functionally important receptor in RMS (especially ARMS) cell lines and tumor tissues. To our knowledge, the present study is the first to verify the antitumor effects of c-Met inhibitor SU11274 in RMS cells. However, additional *in vivo *studies are needed to determine whether inhibiting the phosphorylation of c-Met by SU11274 is a viable therapeutic agent for RMS.

## List of abbreviations

RMS: rhabdomyosarcoma; ERMS: embryonal rhabdomyosarcoma; ARMS: alveolar rhabdomyosarcoma; RTK: receptor tyrosine kinases; HGF/SF: hephotocyte growth factor/scatter factor; mTOR: mammalian target of rapamycin; DMEM: Dulbecco's Modified Eagle Medium; CBB: Coomassie Brilliant Blue; DMSO: dimethyl sulfoxide; ECL: enhanced chemiluminescence; EGFR: epidermal growth factor receptor; IGF-IR: insulin growth factor-I receptor; PI3K: phosphoinositide-3-kinase; MAPK: mitogen activated protein kinase.

## Competing interests

The authors declare that they have no competing interests.

## Authors' contributions

JH, SJD and SHH select the research topic, JH conducts the majority of the experiments, statistical analysis and writes up the draft of the manuscript. LS and JZ conduct the pathological examination. LG and JW conduct trans-well assay. JD, YL, and JB provide technique assistance. SHH and SJD conceive the study project, organize the whole study process, provide financial support, edit and finalize the manuscript. All authors have read and approved the final manuscript.
